# ChimericSeq: An open-source, user-friendly interface for analyzing NGS data to identify and characterize viral-host chimeric sequences

**DOI:** 10.1371/journal.pone.0182843

**Published:** 2017-08-22

**Authors:** Fwu-Shan Shieh, Patrick Jongeneel, Jamin D. Steffen, Selena Lin, Surbhi Jain, Wei Song, Ying-Hsiu Su

**Affiliations:** 1 JBS Science, Inc., Doylestown, Pennsylvania, United States of America; 2 U-Screen Dx Inc., Doylestown, Pennsylvania, United States of America; 3 Drexel University College of Medicine, Philadelphia, Pennsylvania, United States of America; 4 The Baruch S. Blumberg Institute, Doylestown, Pennsylvania, United States of America; Institut de Biologie Paris Seine, FRANCE

## Abstract

Identification of viral integration sites has been important in understanding the pathogenesis and progression of diseases associated with particular viral infections. The advent of next-generation sequencing (NGS) has enabled researchers to understand the impact that viral integration has on the host, such as tumorigenesis. Current computational methods to analyze NGS data of virus-host junction sites have been limited in terms of their accessibility to a broad user base. In this study, we developed a software application (named ChimericSeq), that is the first program of its kind to offer a graphical user interface, compatibility with both Windows and Mac operating systems, and optimized for effectively identifying and annotating virus-host chimeric reads within NGS data. In addition, ChimericSeq’s pipeline implements custom filtering to remove artifacts and detect reads with quantitative analytical reporting to provide functional significance to discovered integration sites. The improved accessibility of ChimericSeq through a GUI interface in both Windows and Mac has potential to expand NGS analytical support to a broader spectrum of the scientific community.

## Introduction

Many viruses, such as the hepatitis B virus (HBV), integrate into host genomes causing genomic disruption and instability [1,2]. These integration events may facilitate progression of consequential disease states, such as cancer, suggesting the identification and characterization of virus-host integration sites can provide important insights into tumorigenesis [3,4]. With an increasing amount of next generation sequencing (NGS) data being generated, efficient and sensitive tools that are accessible to a broader user base are needed to facilitate NGS data analysis [5,6]. However, ongoing investigation of virus-host integration sites has been limited by the scopes of currently available tools for NGS analysis of chimeric reads.

For instance, to the best of our knowledge, most currently available programs require command line arguments and a Linux based knowledge, which limits accessibility for Mac-users and biologists who are not familiar with Linux based distributions. Tools such as VirusSeq [7] can detect viral integration sites in whole-transcriptome sequencing (RNA-Seq) data, but focus on the human genome and a predefined selection of viral genomes. VirusSeq and another similar program, ViralFusionSeq [8], both use alignment algorithms that require high CPU resources, thereby taking a significant amount of time to process whole genome sequencing (WGS) data. Although newer tools such as VirusFinder [9] and Virus-Clip [10] have improved upon accuracy and speed in their analysis of WGS data, all currently available programs still require command line arguments and a Linux based environment. In this study, we developed a program, ChimericSeq, which can efficiently and intuitively identify and annotate virus-host junction sequences within NGS data, in both Windows and Mac environments.

ChimericSeq can identify chimeric junction sequences between two organisms within NGS data including WGS, RNA-seq, and targeted sequencing (TS). This software application expands upon the current standards of annotation, providing in depth qualitative and quantitative analytical reporting, such as the ability to provide position and orientation information of each identified sequence, melting temperature (Tm) analysis, gene information, and homology analysis. These analyses reveal important biological features in studying the pathogenesis of diseases such as cancer. As the first in its class to provide users with an intuitive graphical user interface (GUI) and compatibility with both Windows and Mac operating systems, ChimericSeq, provides a more accessible way to facilitate the identification of viral integration sites.

## Materials and methods

### Preparation of synthetic integration reads

Fifteen independently simulated NGS datasets were synthesized from the reference human genome sequence (hg38) [11] and designed to contain a subset of reads with HBV fragments of various lengths. For each dataset, WGS read simulations were initially created using the third party tool, wgsim (https://github.com/lh3/wgsim). Then, for each simulation, various random fragments of the HBV genome were inserted into a fraction of the reads at random. The synthesized NGS reads were created such that, in each simulation, 18% of reads contained random fragments of the HBV genome. The HBV fragments were evenly distributed in set lengths of 15, 25, 35, 50, 75, or 100 base pairs (bp) of HBV. Within the category, reads were evenly distributed in which HBV was either joined at the 5’ terminus, at the 3’ terminus, or in the center of the 100bp simulated hg38 read. No HBV insertion was used as a control to gauge specificity. Datasets were also generated in a similar fashion to create reads with HIV DNA fragments of various lengths inserted into human genomic DNA, as well as two separate genomic DNA regions to create an artificial fusion event. Datasets of these reads can be obtained at the following link: https://github.com/JBSScience/ChimericSeq.

### Study subjects

The HCC tissue samples used in this study were obtained with written informed consent from patients at the National Cheng-Kung University Medical Center, Taiwan, in accordance with the guidelines of the Institutional Review Board, from the office of regulatory research compliance at Drexel University College of Medicine. The IRB ID is 1203001035 (19321). The IRB specifically approved this study.

### Preparation of NGS reads of clinical samples

HCC tissue DNA was isolated from HBV-infected patient samples using the Qiagen DNeasy Tissue kit (Valencia, CA) according to the manufacturer’s instructions. Tissue DNA was fragmented by sonication and subjected to next-generation sequencing (NGS) library DNA preparation as previously described [12]. NGS was performed to generate 150 bp paired-end reads on the Illumina MiSeq platform (Penn State Hershey Genomics Sciences Facility at Penn State College of Medicine, Hershey, PA). Data was obtained from the Illumina Miseq platform. The clinical patient data used in this study can be freely accessed through the NCBI Sequence Read Archive (SRA), by referencing the BioSample accessions SAMN06290439, SAMN06290437, and SAMN06826930.

### Parameters used for ChimericSeq

ChimericSeq’s alignment algorithm is optimized for an efficient and accurate alignment of the reads. Each alignment uses Bowtie2’s Burrows-Wheeler Transform alignment algorithm using a modified version of Bowtie2’s very-sensitive-local-alignment mode. The parameters for this alignment mode are as follows: -D 20 -R 3 -N 1 -L 20 -i S,1,0.50. These parameters are further explained in the Bowtie2 manual [13]. Briefly, setting N to 1 increases the sensitivity and allows for a robust alignment when dealing with variants, as it deals with the number of mismatches that can occur during multi-seed alignment. L is the length of the seed, where smaller lengths mean increased sensitivity of the alignment. A length of 20 was chosen as it is an optimal balance between sensitivity and specificity. The “i” parameter is the seed interval function, where initial breakdown of the read for seeding alignment occurs.

### Validation of chimeric NGS reads by PCR

HBV chimeric reads generated from HBV-HCC tissue DNA were validated by designing specific PCR assays to amplify a region including the integration site. The reactions contained Hotstart Taq Polymerase (Qiagen, Valencia, CA), specifically designed primers (see S1 Table), and the original genomic tissue DNA. The PCR products that amplified from each assay were viewed on a 2.2% FlashGel DNA Cassette (Lonza Group, Basel, Switzerland). The PCR product was subsequently validated by Sanger sequencing at the NAPcore facility at the Joseph Stokes Jr. Research Institute (Philadelphia, PA).

## Results

### Implementation of ChimericSeq

ChimericSeq was developed to detect integration sites between the genomes of any two organisms, and is meant to reach the broader scientific community. An intuitive GUI allows users who are not familiar with Linux based distributions to operate the program with point and click. There is no need for editing configuration files or source deconstruction, and no prior bash scripting knowledge is required to run the program. In order to make the program efficient and robust, ChimericSeq was designed to take advantage of Bowtie2 by implementing all required algorithms for alignment internally and relying on the use of only three dependencies: Bowtie2, Python and Perl. In comparison to other similar programs such as Virus-Clip, ViralFusionSeq, and VirusFinder2, ChimericSeq requires shorter setup time because of fewer dependencies (Table 1). In addition to the easy installation, ChimericSeq enables error-checking of processing parameters, provides analytical annotation features, and is able to deal with memory limitation via file-splitting.

**Table 1:** Comparison of required third-party dependencies.

Requirement	Chimeric-Seq	VirusSeq	Virus-Clip	ViralFusion-Seq	Virus-Finder2
ANNOVAR			X		
Bash		X	X	X	X
BEDtools				X	
BLAST				X	
BLAST+			X		X
BLAT					X
Bowtie2	X				X
BWA			X	X	X
CAP3				X	
CREST					X
GATK					X
iCORN					X
Java					X
Linux		X	X	X	X
Mosaik		X			
Perl	X	X	X	X*	X*
Python	X				X
Samtools			X	X	X
SSAKE				X	
SVDetect					X
Trinty					X

*Denotes that additional libraries must be installed into the Perl distribution.

**Fig 1:**
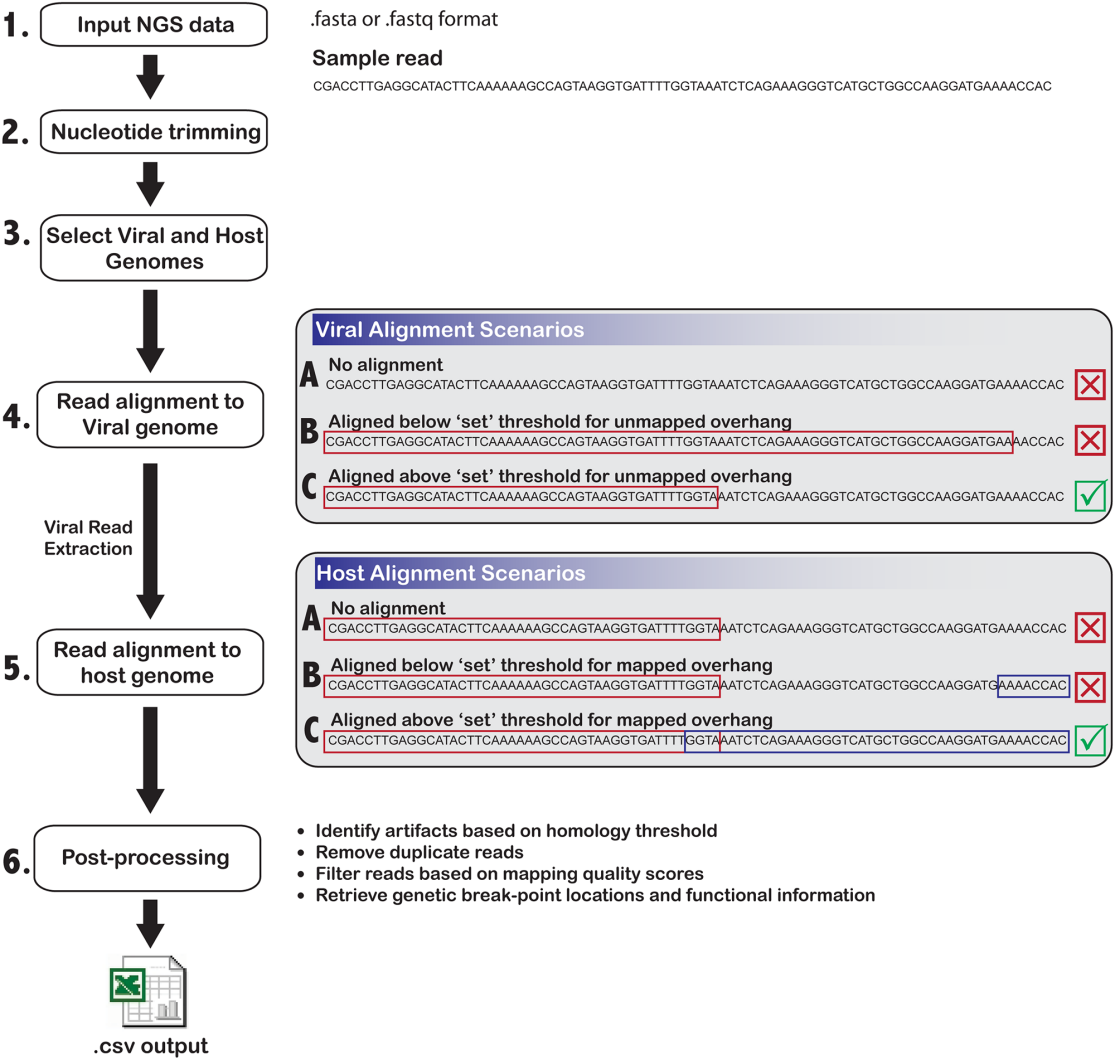
Schematic overview of the ChimericSeq workflow. Input NGS reads are manually loaded through a graphical interface, followed by user-determined 5’ and 3’ end trimming. Host and viral genomes and indices must be identified, if not otherwise already loaded. Next, the identification phase aligns each read to the specified viral genome, extracts these aligned reads, and then aligns the reads to the host genome. The identification phase is further broken down to describe potential scenarios, where 1) the read has no alignment to the viral genome, and is thus discarded, as indicated by the “X”, 2) the read has alignment to the viral genome, however the unmapped region’s length is lower than the threshold set by the program (or user), and is thus discarded, and 3) the read has alignment to the viral genome and has sufficient unmapped overhang for alignment to the host genome, and is extracted (as indicated by the checkmark). The extracted reads are then subjected to Bowtie2 alignment to the host genome, following similar scenarios as depicted. The identified chimeric reads are then passed to the post processing phase, which includes steps to filter out artifacts and annotate integration sites with functional information such as gene breakpoint location. Finally, reads are presented through the program interface and saved to accessible output files.

The overall workflow of the program is shown in Fig 1. To detect chimeric reads, individual or mate paired NGS reads in Fastq format are input into ChimericSeq. If needed, ChimericSeq will perform nucleotide trimming by removing a user-determined amount of bases from the 5’ and/or 3’ end of each read before alignment. Host and viral genomes of interest must next be supplied, as well as input of the species-specific genome reference annotation file in general transfer format (gtf). If the host and viral genome index files have not been built, the user must either use the ‘build’ option to create index files or download existing index files, for instance, files from the JBS ChimericSeq website (http://www.jbs-science.com/ChimericSeq.php). Each read within the input file(s) is first aligned to the viral reference of choice using Bowtie2’s Burrows-Wheeler Transform alignment algorithm [13]. Using local alignment mode, reads that align to the viral reference genome and contain an unmapped portion above a threshold length are extracted (Fig 1), and all other reads are discarded. This threshold can be set by the user and is needed to ensure a more accurate alignment to the host genome. The extracted reads are then aligned to the host genome using the Bowtie2 alignment algorithm once again in local mode. Finally, the reads that contained partially aligned regions to the host are extracted, while those that did not are discarded.

Should any read be non-uniquely mapped, ChimericSeq follows the convention set by Bowtie2’s default behavior and returns the mapping with the highest quality score. The alignment data is subsequently passed into the post processing stage. This stage includes artifact detection by computing analysis on homologous regions, filtering reads using mapping quality scores, identifying orientation, removing multi-mapped reads and reads with a large unmapped portion, and passing reads through a sorted tree structure (as detailed in the next section) in order to highlight and/or filter by function or target of interest(s). The criteria of the post-processing step can be defined by the user under the options>configurations tab. Processed reads are then annotated and presented to the user via the GUI. The final data can be saved as a series of csv files, allowing the user to access identified unique integration sites in Microsoft Excel or similar applications (such as Google Sheets).

**Fig 2:**
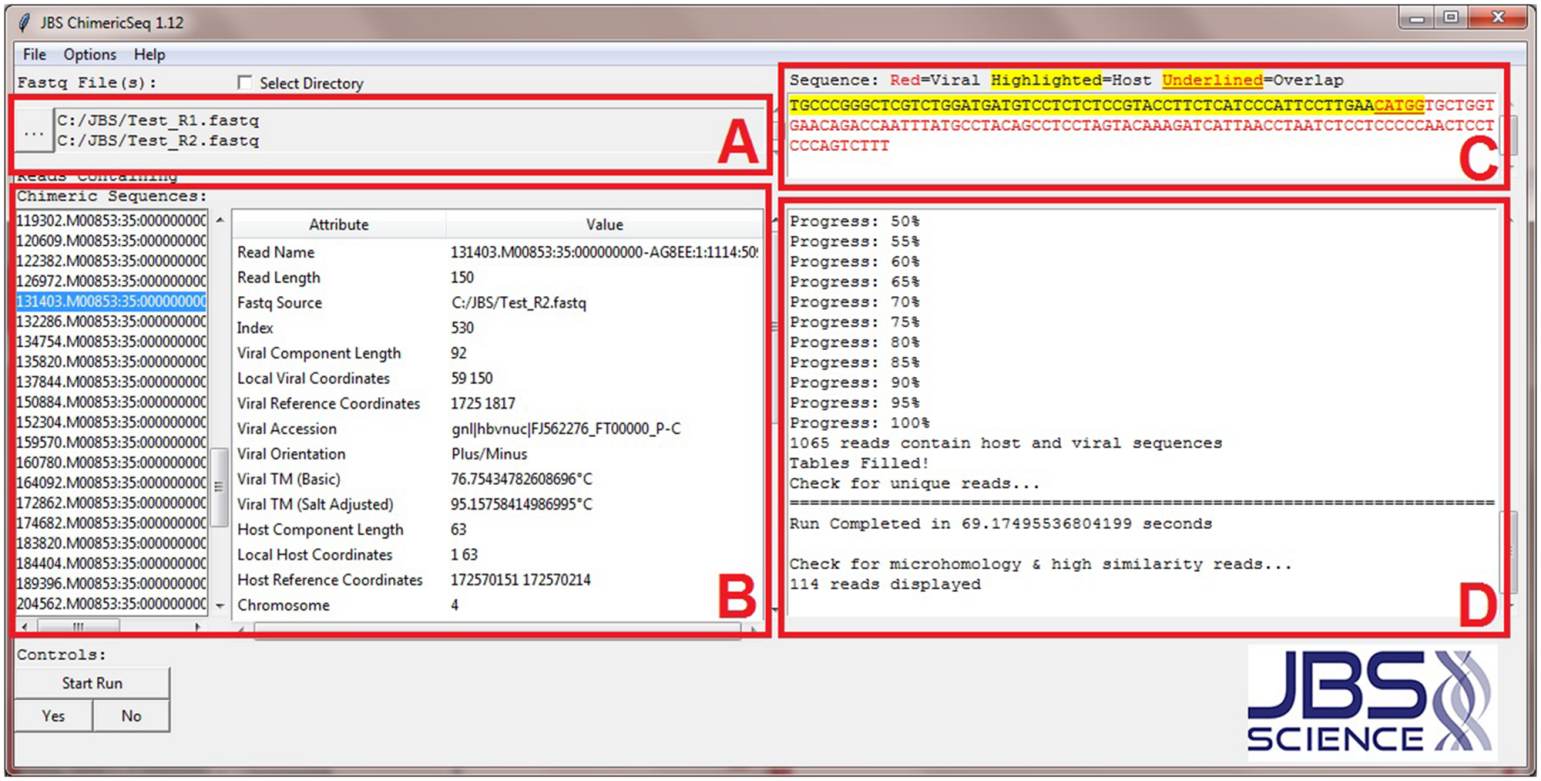
Description of ChimericSeq’s interactive, graphical user interface (GUI). (A) Sequence data of host, virus, and sample NGS reads in fastq format is loaded into the program. (B) Reads containing integration sites are displayed in a column format. Analytical data associated with the selected read is displayed within the table. (C) The selected read is visualized to highlight different segments and overlap. (D) Interactive display that communicates questions to the user and also provides logistical information about the run.

A unique feature of ChimericSeq is in its ability to provide in depth annotation of processed reads identified as unique integration sites. ChimericSeq facilitates the annotation by building a sorted tree structure of all gene positions and features using an input human genome reference annotation file in general transfer format (gtf). This genome reference annotation file contains host gene information and can be downloaded from ensembl.org (http://www.ensembl.org/info/data/ftp/index.html) or from the link provided on the JBS website (http://www.jbs-science.com/ChimericSeq.php). By using this sorted tree structure to annotate the data, the process can be efficiently streamlined. Upon identification of each chimeric read, the annotation data is obtained from this sorted tree structure and a comma-separated values (csv) file of annotated information corresponding to each identified chimeric read is generated with completion of the run. As a result, after analysis, all identified chimeric reads are displayed in a list (Fig 2B). Each read can be selected, and a visual display of the read derived from the annotation data file is shown (Fig 2C), with distinct sequence information of the host and viral components. Sequence information such as length, local coordinates, reference coordinates, and chromosome breakpoints are also displayed. Furthermore, this annotation provides the information to determine whether the integration site lies within a gene, or upstream or downstream of a gene. The annotation reports if the integration site is located on a known exon, transcript, start or stop codon, etc. Additional attributes include the length and melting temperature (Tm) of the host, virus, and overlapping segments (both basic and salt adjusted via user defined data). Segment orientation and mapping quality are also reported within the interface. All of this data can be saved to a central output csv file upon the user’s request, and opened in Excel. The number of unique reads and supporting reads per sequence can also be generated via the output.

**Fig 3:**
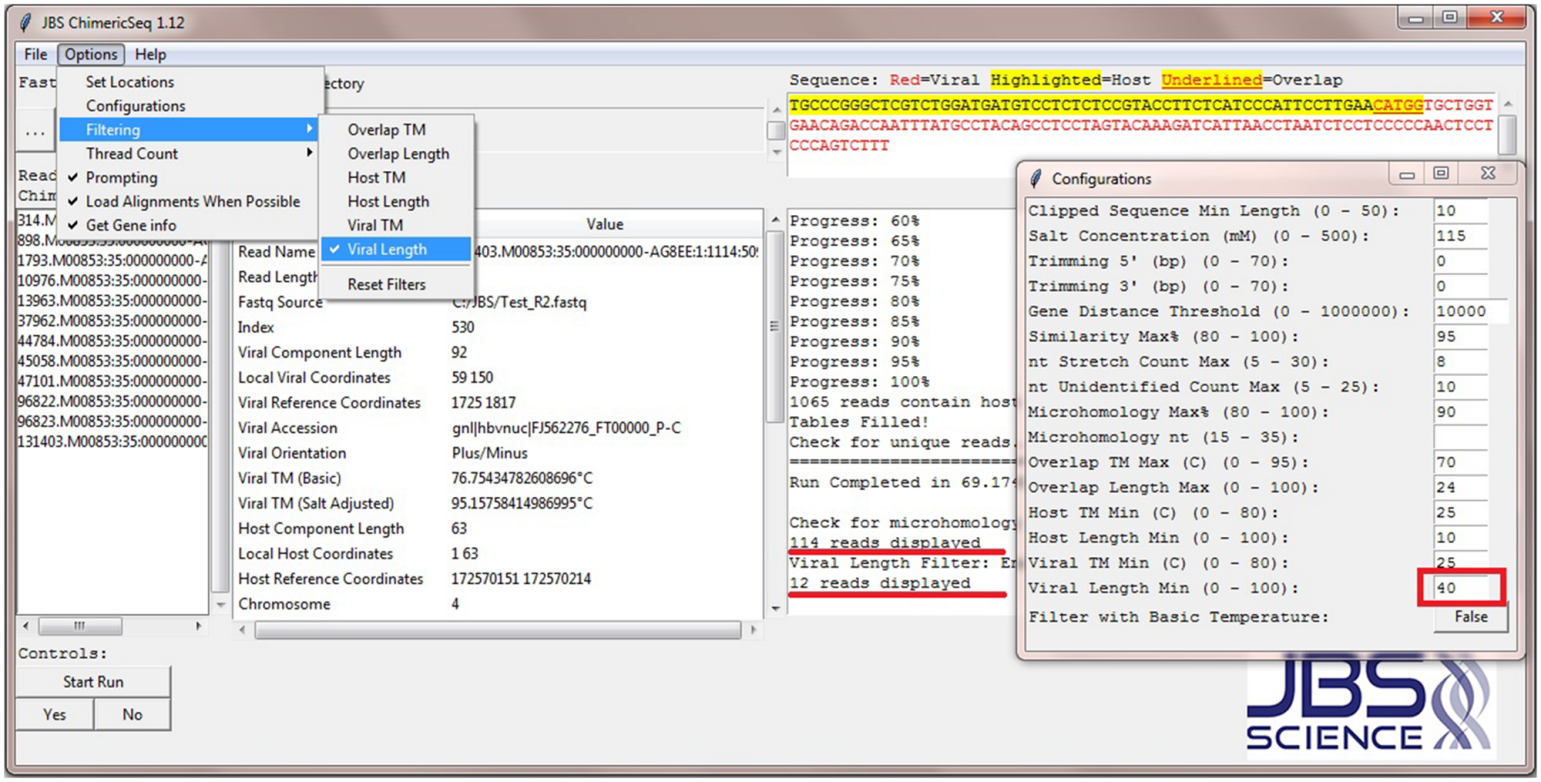
ChimericSeq’s configurations window and filtering. The configurations window is used to set up process parameters. Each parameter’s value range has been specified, so the user does not have to remember all the details. The user may refine the reads selection via changing the host/viral setting(s) and enabling filter(s).

Unlike the Linux command line or scripting file handling, ChimericSeq’s graphical user interface enables the user to configure process parameters easily in the options>configurations tab. ChimericSeq also provides error checking for process parameters. If the user configures a process parameter with a value out of the specified range or incorrect format, a pre-defined value will be used to avoid system error. During the post-processing, the user may further refine the reads selection via changing the host/viral settings (“Overlap TM Max”, “Overlap Length Max”, “Host TM Min”, “Host Length Min”, “Viral TM Min”, and “Viral Length Min”) and enabling corresponding filter(s) (See S1 Table for descriptions of filters). For instance, the number of selected reads from the data accessible file (Sample reads from http://www.jbs-science.com/ChimericSeq.php) is reduced from 114 to 12 when the “Viral Length Min” is set to 40 and “Viral Length” filter is enabled (Fig 3).

Users working on either Windows or Mac systems may encounter memory limitation when processing files containing massive amounts of data. In order to resolve this memory issue, ChimericSeq uses Biopython’s FastqGeneralIterator [14] to provide a functionality to split large paired files into smaller size (1,000,000 reads) paired files before processing.

### Identification of HBV integration sites

As an initial assessment of ChimericSeq’s capability in identifying chimeric reads, we tested a set of known reads that were randomly generated *in silico*. Synthetic NGS datasets were generated to contain random HBV-host chimeric sequences as positive controls, and random HIV-host and host-host chimeric sequences as negative controls (described in Methods).

Table 2 shows the comparison of the performance of ChimericSeq with two similar programs, Virus-Clip and ViralFusionSeq. VirusFinder2 was included in initial analysis of program requirements, but despite several attempts using different environments, it could not be run due to technical errors during setup. VirusSeq was not included in this comparison because it does not report the breakpoints between the host and virus. This program is rather designed for discovery of integration events given two known organisms. Thus, we compare ChimericSeq with ViralFusionSeq and Virus-Clip since they are the most similar in terms of the functions of the software. Three simulations were performed on artificial, random generated reads with varying HBV insertion lengths, as described in Materials and Methods.

**Table 2:** Percent detection of HBV integration sites with defined lengths of viral DNA insertion.

HBV Insertion length (bp)	0	15	25	35	50	75	100	Run Time (seconds)
**ChimericSeq**	0	0	100	100	100	100	100	1.9 ± 0.1
**Virus-Clip**	0	0	0	97 ± 3	100	82 ± 20	80 ± 23	35 ± 7
**ViralFusionSeq**	0	0	0	44 ± 20	62 ± 8	84 ± 3	84 ± 3	333 ± 58

ChimericSeq (using the Bowtie2 default seed length setting of 20 nt) successfully detected 100 percent of chimeric reads in the synthetic NGS dataset when the viral inserted fragment was 25 base pairs or higher. As expected, reads containing an HBV insert size of 15 base pairs could not be detected since they are shorter than the default seed length of 20. The total runtime for each program (Table 2) is also reported, indicating the amount of time needed for a program to produce its output on a dataset from the time it started. ChimericSeq averaged 1.9 seconds of total runtime for each simulated dataset. Virus-Clip with its default setting was not able to detect any of the reads containing 15 or 25 bp fragments and had difficulty detecting HBV integrated reads larger than 50 bp, detecting on average 82.2% and 80% of the reads containing 75 and 100 bp HBV, respectively. It was, however, able to detect 97.7% of the reads containing 35 bp fragments and 100% of 50 bp fragments. This loss in sensitivity with larger reads could be due to the seed length, as we have also observed with ChimericSeq that performance with the 75 and 100 bp HBV set decreases to 73.3% when Bowtie’s seed length is increased from 20 to 25. The average runtime per dataset for Virus-Clip was 35.6 seconds, which was 18.7 times longer than ChimericSeq’s runtime. ViralFusionSeq, with its default setting, was also not able to detect any 15 or 25 bp fragments, and had difficulty detecting reads with HBV fragments 35 and 50 bp in length, detecting 44.4% and 62.2% respectively. It was able to discover 84.4% of reads containing 75 and 100 bp fragments. ViralFusionSeq’s average runtime was 333 seconds, 175 times longer than ChimericSeq.

Since ChimericSeq and Virus-Clip both showed high sensitivity with significantly shorter run times, we further tested non-HBV integrated DNA test sets in both programs to evaluate the false positive rate. Reads were generated containing HIV DNA integrated randomly into human genomic DNA sequences, as well as human DNA fusion reads. Both ChimericSeq and Virus-Clip were tested on these datasets with HBV used as the viral genome to search. As expected, no reads were identified, indicating no false positives were detected by ChimericSeq or Virus-Clip from the randomly generated, *in silico* negative control data set.

### Detection of integrated HBV from HBV-HCC NGS data

To test the performance of ChimericSeq with clinical specimen data, we analyzed NGS reads obtained from human HCC tissue DNA. These NGS reads were used to identify major HCC associated HBV integration junctions from carcinogenesis-related clonal expansion, through the presence of multiple, unique reads of a particular chimeric sequence. Each sample was obtained via the Illumina Miseq platform from patients with a known HBV infection. A summary of results on the processing of these samples in comparison to other similar programs is listed in Table 3. In all cases, the total runtime of ChimericSeq was significantly faster, averaging at least 5 times faster than Virus-Clip, and over 100 times faster than ViralFusionSeq. In some cases, we have seen how ViralFusionSeq fails upon execution, a setback also observed by others [10].

**Table 3:** Evaluation of integration sites from NGS data of HBV-infected patients.

Patient / SRA#	Program	Run Time (seconds)	Chimeric Reads Detected	Unique Chimeric Reads Detected
**1**	ViralFusionSeq	6,105	466	120*
SRS1954054	Virus-Clip	141	2,253	565*
	ChimericSeq	23	3,264	1,062
**2**	ViralFusionSeq	1,306	97	18*
SRS1954056	Virus-Clip	62	337	115*
	ChimericSeq	7	413	180
**3**	ViralFusionSeq	6648	5222	901*
SRS2140524	Virus-Clip	180	7319	2145*
	ChimericSeq	33	4470	2476

*Denotes the data was not provided as an inherent function of the software, and was manually extracted

To validate the detected NGS reads, we designed specific PCR based assays for the major chimeric sequence of each dataset (or integration site with the most abundant number of supporting reads). False positives from NGS reads are often generated experimentally through library preparation (involving PCR amplification) and the inherited sequencing error (the range of 1–5%) of next generation sequencing [15]. In order to avoid false positives generated experimentally in our validation of computational performance, we focused our validation efforts around the chimeric read containing the most unique supporting reads (i.e. the major unique chimeric sequence). The PCR assays were designed to target on both the HBV and human side of the chimeric sequence to generate an amplicon that includes the integration junction sequence (S2 Table). To further confirm the junction sequences, the amplicon products were purified and subjected to Sanger sequencing as indicated in the chromatograms of sequencing data. For all three patient samples, we were able to detect the correct amplicon size directly from the source tissue DNA (S1 Fig). As a negative control we also tested DNA isolated from the HepG2 cell line, which is human genomic DNA.

To illustrate the output of data analysis, the unique reads generated for patient #2 by ChimericSeq, Virus-Clip, and ViralFusionSeq are shown in S2, S3 and S4 Figs respectively. Note, ChimericSeq’s genetic annotation (displayed in greater detail in the output.csv files) of identified chimeric reads is an important built-in feature that is significant in determining the integration site identified. For instance, ChimericSeq displayed the Cycline E1 (CCNE1) gene integration for the major chimeric sequence of patient #2. This is very important to understand the potential role of HBV integration. CCNE1 is a known gene associated with liver carcinogenesis and has been found in other HBV-associated HCC tissue DNA as a driver for HCC [3]. The true reads associated with the major chimeric sequence (CCNE1 gene integration) are sectioned off for comparison (labeled reads 1–8 in S2, S3 and S4 Figs). ChimericSeq does not identify the 8^th^ read in this set because the human genomic portion is only 19 base pairs long in this read, and Bowtie2 in its very sensitive alignment mode (see Methods) filters out reads below 20 base pairs long.

### ChimericSeq’s analysis of large datasets and varying NGS types

Next, we tested the performance of ChimericSeq, on a large data set. ChimericSeq was used to analyze 13 million reads of simulated human WGS reads integrated with the HPV-16 virus provided by VirusFinder2 [9]. This was a simulated dataset of reads derived from a single integration of a mutated HPV-16 virus within chromosome 1 of the human genome. Using both the reference genomes identified by VirusFinder2, the mutated HPV-16 virus reference genome (GI: 310698439) and the human genome UCSC hg38, ChimericSeq was able to detect and fully characterize the integration event in chromosome 1, with a total processing time of just 33.2 minutes. This showed once again that ChimericSeq was efficient in its detection process.

## Discussion

This study describes the development and evaluation of ChimericSeq, a user-friendly program for the identification and characterization of integration sites in NGS data. To our knowledge, this is the first program of its kind to offer support in an intuitive GUI for both Windows and Mac. ChimericSeq was built to handle all analytical features with optimized in-house algorithms, and limits its third party requirements to Bowtie2 and Bowtie2’s interpreters, Python and Perl, for read alignment. The reduction of computational dependencies and use of Bowtie2’s implementation of algorithms internally allows for a more efficient performance. These improvements were demonstrated in a comparison between ChimericSeq and other current programs to identify chimeric reads using a synthetic data set we created, NGS data generated from three HCC tissue DNA samples, and a WGS dataset provided by VirusFinder2 containing an HPV integration event [9].

While current software is available to analyze NGS data for viral integration sites, prior knowledge of programming and familiarity with a Linux-based environment are often needed to perform analysis. ChimericSeq takes a meaningful part in strengthening the bridge between genomic analytics and translational science, by bringing forth an easy-to-use GUI with quick setup. Furthermore, the functions for integration site detection, multiplatform support, and an extensive annotation system are also built within the software. As a result, in addition to identifying integration sites in NGS data, ChimericSeq offers features such as read visualization, sequence breakdown specifics (including salt adjusted melt temperatures), and insight into genomic overlap and characterization of the reads, for example whether the integration site is in a promoter region for a specific gene. The ability to see integration related information next to each read will likely be of interest to researchers in the field of biology, (particularly cancer biology) and can be seen in part from the output generated from ChimericSeq (S2 Fig) on the unique reads for Patient #2. ChimericSeq generates well-annotated reads in Excel format that allowed us to determine easily that there is a major integration site in the CCNE1 gene (which we confirmed by Sanger sequencing). Major chimeric sequences (or integration sites containing abundant supporting reads) are useful in providing compelling evidence for a particular unique chimeric read. In the S2, S3 and S4 Figs we have manually highlighted these reads for the CCNE1 integration to make the comparison apparent. While both programs can successfully identify the true chimeric sequence for this patient sample, ChimericSeq makes the analysis and characterization much faster and more intuitive. ChimericSeq does not pick up the 8^th^ read that Virus-Clip does, but this is due to the set seed length. Since the viral length in this read is 19 bp long and ChimericSeq by default uses a minimum 20 bp seed length, it is missed. Setting the seed length lower would detect reads like this at the expense of more computation time, while increasing the length may miss true integration reads.

ChimericSeq’s pipeline features advanced processing abilities, such as the use of genomic annotation tables for the identification of nearby genes and functional regions to the integration site, segment orientation, and melt temperature of the overlapping sequences. Filtering mechanisms (as detailed in S1 Table) allow users to not only screen for genomic targets of interest, but also allow ChimericSeq to detect artifacts based on homology length and percentage (as referred to as “microhomology”) and the minimum length setting for both virus and host sequences as alignment-based quality checks. For instance, reads with a high degree of “microhomology” (S1 Table) may be considered artifacts by the user, and thus discarded. Reads with ambiguous multi-mappings may also be discarded. These post-processing steps provide greater functional significance associated with the detected reads. Note, these alignment-based quality checks are only for optimizing the mapping quality, and not for validation. Validation of chimeric reads require additional experimental approaches on the originating DNA sample, such as PCR to confirm the existence of a true integrated sequence (examples shown in S1 Fig).

Our comparison with similar programs (Tables 1–3) suggests ChimericSeq analyzes and detects integration sites in reads more quickly. Improvements in speed could be attributed to streamlined in-house algorithms which define processing and annotation steps, as well as ChimericSeq’s use of more efficient algorithms, notably Bowtie2’s FM-indexing approach based on the Burrows Wheeler Transform [13]. Bowtie2 processes data efficiently because it integrates all required algorithms (no 3^rd^ party calling is needed), thereby optimizing memory usage and reducing processing times that ultimately result in decreased communication time spent between dependent tools; this implementation has become increasingly popular in the field [16]. The algorithm used by ChimericSeq, Bowtie2, was optimized for an efficient alignment of the reads, as compared to bwa and bwa-sw algorithms [17], or NCBI’s BLAST algorithm; the latter accounts for a significant portion of its runtime. By avoiding this, ChimericSeq saves computational hours and allows for practical application on WGS datasets.

In the synthetic data set, all reads containing HBV fragments were known before testing. The randomized integration technique described was used because it closely mirrors the seemingly random behavior of HBV integration in nature [18]. Encouragingly, ChimericSeq could identify reads as short as 25 bp and as long as 100 bp from the positive control data set. Although we did not detect false positives from the negative control data set containing random variations of HIV-host integrated or host-host integrated (fusion events) sequences when the input search virus was set to the HBV genome sequence, it is possible that host regions highly homologous to HBV could be detected in the experimental data sets. In the same manner, host only reads could be detected if they contain a stretch of HBV homologous DNA of at least “20 bp” or “90% overlapping” (or whatever the limit is adjusted to in the “microhomology” setting). To better control for false positive or false negative detection, ChimericSeq has several parameter settings (such as a settings for “microhomology” and “minimum length” for host and virus, detailed in S1 Table) that can be adjusted to allow users to customize the settings based on their experimental needs.

While we have primarily focused on using data with HBV integration, ChimericSeq’s versatility in detecting fusion events can be expanded to any organism with a reference, and can also make use of large databases of references within a single run. Further development of ChimericSeq is in progress to expand its abilities to be run on high performance computing clusters (in order to facilitate more sophisticated data mining characterization techniques of integration events) and to implement read-pair analysis to help control for false positives, as demonstrated by recent literature [19]. This program is available for download and features a tutorial covering its full capabilities at our website, http://www.jbs-science.com/ChimericSeq.php.

## Supporting information

S1 Table Description of ChimericSeq filtering configurations. These filtering mechanisms may be adjusted in the ChimericSeq software under **Options>Configurations**.
(DOCX)

S2 Table Primers used to confirm PCR reactions. For the Major integration sequence: lower-case represents HBV DNA, upper-case represents human genomic DNA, and bold represents the overlap. The gray highlight indicates where the primers target. Target gene region indicates the integrated HBV is in the mentioned gene or within 100,000bp of the gene.
(DOCX)

S1 Fig Sanger sequencing validation of NGS identified HBV chimeric reads from HBV-HCC tissue DNA. Tissue DNA from patients was subjected to PCR amplification using unique primers of the major junction sequences identified from NGS analysis. An HBV-enriched tissue library DNA was used as the positive control (+) and DNA from HepG2 cells was used as the negative control (-). The original tissue DNA (A33K, S44K, and A34K) was tested to confirm correct amplicon size, and the amplicon from each sample was Sanger sequenced. The depicted chromatogram contains the chimeric sequence selected with a black box (lower panel). Lower case sequences represent HBV DNA. Underlined and capitalized sequences represent human DNA. Underlined, lower case, and bold sequences represent overlapping human and HBV sequences.
(DOCX)

S2 Fig ChimericSeq output of unique reads for patient 2. (PDF)

S3 Fig VirusClip output of unique reads for patient 2. (PDF)

S4 Fig ViralFusionSeq output of unique reads for patient 2. (PDF)
