# Supplementary material for: ChimericSeq: An open-source, user-friendly interface for analyzing NGS data to identify and characterize viral-host chimeric sequences
Source: PLoS One. 2017 Aug 22;12(8):e0182843. doi: 10.1371/journal.pone.0182843 (PMC5567911; doi:10.1371/journal.pone.0182843)
Supplement: S1 Table — These filtering mechanisms may be adjusted in the ChimericSeq software under Options>Configurations. (DOCX) [file pone.0182843.s001.docx]

| **Paramemter** | **Allowable range** | **Default setting** | **Description** |
| --- | --- | --- | --- |
| Clipped Sequence Min Length | 0-50 nucleotides | 10 nucleotides | When the viral portion is aligned to the reference, this program looks for reads that aligned only partially to the viral reference file. This field is the threshold length of the unaligned portion. For example, a 50 base pair read with the base pairs 7-45 aligning to the viral reference file would have clipped sequences of lengths 6 and 5. This read would not qualify to be aligned to human under the default value (10). |
| Salt Concentration | 0-500mM | 115mM | This is the value of the salt concentration for determining the salt adjusted melt temps of each DNA segment (host, viral, and overlap), as given by the DNA melt temperature formulas. This can be useful in primer design. |
| Trimming 5’ | 0-70 nucleotides | 0 | Takes off this number of bases from the 5’ end for trimming the adapter sequences of reads. |
| Trimming 3’ | 0-70 nucleotides | 0 | Takes off this number of bases from the 3’ end for trimming the adapter sequences of reads. |
| Gene Distance Threshold | 0-1,000,000 bases | 10,000 bases | The closest upstream or downstream gene to the overlap of the chimeric integration read (if not inside the gene). |
| Similarity Max% | 80-100% | 95% | When 2 reads contain greater than the set percentage of homologous (or identical) sequence, only 1 read will be retained. |
| Stretch count max | 5-30 nucleotides | 8 nucleotides | When there is a repetitive run in the sequence of the same nucleotide, the read is discarded. |
| Microhomology Max% | 80-100% | 90% | When the identified viral sequence has more than the set maximum percent overlap with the host sequence, the read is discarded. |
| Microhomology | 15-35 nucleotides | 20 nucleotides | When the identified viral sequence has more than the set maximum number of nucleotides with the host sequence, the read is discarded. |
| Overlap Tm Max | 0-95°C | 70°C | Value specifies the maximum overlap melt temperature to help remove artifacts. |
| Overlap Length Max | 0-100 nucleotides | 24 nucleotides | Value specifies the maximum allowed overlap length to help remove artifacts. |
| Host Tm Min | 0-80°C | 25°C | Value specifies the minimum host region melting temperature. |
| Host Length Min | 0-100 nucleotides | 10 nucleotides | Value specifies the minimum host region length. |
| Viral Tm Min | 0-80°C | 25°C | Value specifies the minimum viral region melting temperature. |
| Viral Length Min | 0-100 nucleotides | 10 nucleotides | Value specifies the minimum viral region length. |
