# Supplementary material for: ChimericSeq: An open-source, user-friendly interface for analyzing NGS data to identify and characterize viral-host chimeric sequences
Source: PLoS One. 2017 Aug 22;12(8):e0182843. doi: 10.1371/journal.pone.0182843 (PMC5567911; doi:10.1371/journal.pone.0182843)
Supplement: S2 Table — For the Major integration sequence: lower-case represents HBV DNA, upper-case represents human genomic DNA, and bold represents the overlap. The gray highlight indicates where the primers target. Target gene region indicates the integrated HBV is in the mentioned gene or within 100,000bp of the gene. (DOCX) [file pone.0182843.s002.docx]

| **Patient ID** | **Genome location (human:HBV)** | **Primer Sequence (5’- 3’)**  **(human:HBV)** | **Major Integration site** |
| --- | --- | --- | --- |
| Patient 1 (A33K) | GRCh38.p7, Chr16: 29467756-29467737  HBV: 1792-1811  Target gene region: RP11-345J4.4 | CTTGGCCATTGGCTCTATGT  AATTGGTCTGTTCACCAGCA | actaggaggctgtaggcataaattggtctgttcaccagcaccatgcaactttt**t**TCCGAACCTGTGTACTAAACTGCCTGGGGGCAGCTCTCATCACTGCTGTAGAACAAAGTCCCACATAGAGCCATGGCCAAGAACCAGTTAATAAAA |
| Patient 2 (S44K) | GRCh38p7, Chr19: 29812893-29812873  HBV: 1748-1772  Target gene region: CCNE1 | GATCAGGTTATGAGCTCCGTTG  GGAGATAAGGTTAAAGGTCTTTGTA | gaggagattaggctaaaggtctttgtactaggaggctgtaggcataaattggtctgttcaccagcaccatgc**aac**GGAGCTCATAACCTGATCAGCTTTCTCTTCTTCTCTCTGTTTTTGTCTTGTTGGTGTGTTTCCTTGGGGTCATGG |
| Patient 3 (A34K) | GRCh38p7, Chr5: 1292213-1292195  HBV: 1685-1709  Target gene region: TERT | CTCCCCACAAACTCCCAAG  AACGACCGACCTTGAGGCATACTTC | aacgaccgaccttgaggcatacttcaaagactgtttgtttaaggagtgggaggagttgggggaggagattaggttaaaggtctttgtactaggagg**ctg**CATGGCCGGAAGTCTTACATGTCTTGGGAGTTTGTGGGGAGGGGGTGAAATCGGGACTTCTTCTAGCTGCCCGG |
