# Supplementary material for: ChimericSeq: An open-source, user-friendly interface for analyzing NGS data to identify and characterize viral-host chimeric sequences
Source: PLoS One. 2017 Aug 22;12(8):e0182843. doi: 10.1371/journal.pone.0182843 (PMC5567911; doi:10.1371/journal.pone.0182843)
Supplement: S1 Fig — Tissue DNA from patients was subjected to PCR amplification using unique primers of the major junction sequences identified from NGS analysis. An HBV-enriched tissue library DNA was used as the positive control (+) and DNA from HepG2 cells was used as the negative control (-). The original tissue DNA (A33K, S44K, and A34K) was tested to confirm correct amplicon size, and the amplicon from each sample was Sanger sequenced. The depicted chromatogram contains the chimeric sequence selected with a black box (lower panel). Lower case sequences represent HBV DNA. Underlined and capitalized sequences represent human DNA. Underlined, lower case, and bold sequences represent overlapping human and HBV sequences. (DOCX) [file pone.0182843.s003.docx]

**Patient 1**


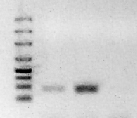

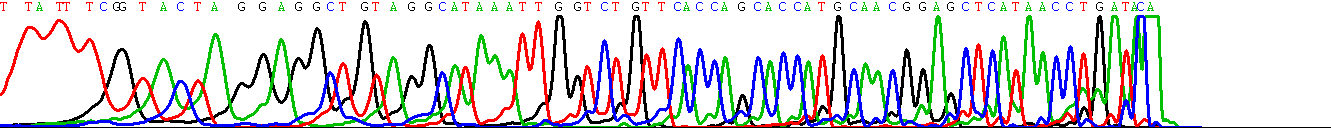


caccatgc**aac**GGAGC

**Patient 2**

200

150

100

50

bp

CAGGTTCGG**a**aaaaag


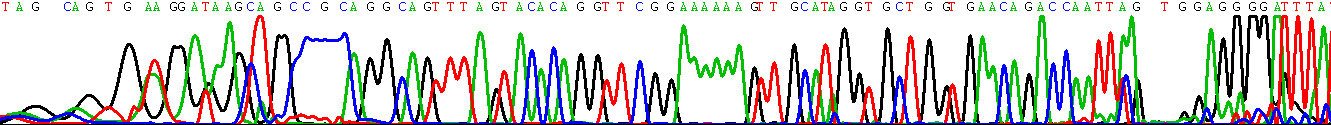

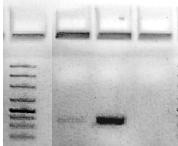


200

150

100

50

bp

**+**

**MW**

**-**

**S44K**

**+**

**MW**

**-**

**A33K**

**Patient 3**


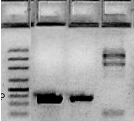


ctaggagg**ctg**CATGG

200

150

100

50

bp


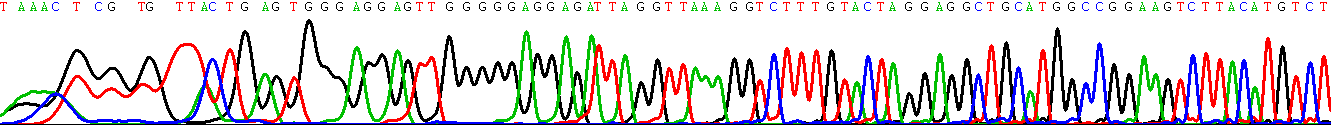


**+**

**MW**

**-**

**S34K**
