# Supplementary material for: ChimericSeq: An open-source, user-friendly interface for analyzing NGS data to identify and characterize viral-host chimeric sequences
Source: PLoS One. 2017 Aug 22;12(8):e0182843. doi: 10.1371/journal.pone.0182843 (PMC5567911; doi:10.1371/journal.pone.0182843)
Supplement: S4 Fig — (PDF) [file pone.0182843.s006.pdf]

M00853:35:000000000-AG8EE:1:2113:17005:13728_Cs_CON_consensus	TCTTCATCTGGATCCTGCAAAAACTAACATGAAGCCACCCCCATGACCCCAAGGAAACACACCAAACAAGACAAAAACAGAGAGAAGAAGAGAAAGCTGATCAGGTTATGAGCTCCGTTGCATGGTGCTGGTGAACAGACCAATTTATGCC	chromosome 19; cyclin E1 (CCNE1) gene
M00853:35:000000000-AG8EE:1:2114:27281:22325_Cs_CON_consensus	TCTTCATCTGGATCCTGCAAAAACTAACATGAAGCCACCCCCATGACCCCAAGGAAACACACCAAACAAGACAAAAACAGAGAGAAGAAGAGAAAGCTGATCAGGTTATGAGCTCCGTTGCATGGTGCTGGTGAACAGACCAATTTATGCC	chromosome 19; cyclin E1 (CCNE1) gene
M00853:35:000000000-AG8EE:1:2112:11821:8209_Cs_CON_consensus	TCTTCATCTGGATCCTGCAAAAACTAACATGAAGCCACCCCCATGACCCCAAGGAAACACACCAAACAAGACAAAAACAGAGAGAAGAAGAGAAAGCTGATCAGGTTATGAGCTCCGTTGCATGGTGCTGGTGAACAGACCAATTTATGCC	chromosome 19; cyclin E1 (CCNE1) gene
M00853:35:000000000-AG8EE:1:1113:18713:19240_Cs_CON_consensus	TCTTCATCTGGATCCTGCAAAAACTAACATGAAGCCACCCCCATGACCCCAAGGAAACACACCAAACAAGACAAAAACAGAGAGAAGAAGAGAAAGCTGATCAGGTTATGAGCTCCGTTGCATGGTGCTGGTGAACAGACCAATTTATGCC	chromosome 19; cyclin E1 (CCNE1) gene
M00853:35:000000000-AG8EE:1:2110:11481:2679_HBV_3215	TCTTCATCTGGATCCTGCAAAAACTAACATGAAGCCACCCCCATGACCCCAAGGAAACACACCAAACAAGACAAAAACAGAGAGAAGAAGAGAAAGCTGATCAGGTTATGAGCTCCGTTGCATGGTGCTGGTGAACAGACCAATTTATGCC	chromosome 19; cyclin E1 (CCNE1) gene
M00853:35:000000000-AG8EE:1:2109:24360:16164_HBV_3215	TCTTCATCTGGATCCTGCAAAAACTAACATGAAGCCACCCCCATGACCCCAAGGAAACACACCAAACAAGACAAAAACAGAGAGAAGAAGAGAAAGCTGATCAGGTTATGAGCTCCGTTGCATGGTGCTGGTGAACAGACCAATTTATGCC	chromosome 19; cyclin E1 (CCNE1) gene
M00853:35:000000000-AG8EE:1:1105:4560:21104_HBV_3215	TCTTCATCTGGATCCTGCAAAAACTAACATGAAGCCACCCCCATGACTCCAAGGAAACACACCAAACAAGACAAAAACAGAGAGAAGAAGAGAAAGCTGATCAGGTTATGAGCTCCGTTGCATGGTGCTGGTGAACAGACCAATTTATGCC	chromosome 19; cyclin E1 (CCNE1) gene
M00853:35:000000000-AG8EE:1:1105:10131:4397_Cs_CON_consensus	TCTTCATCTGGATCCTGCAAAAACTAACATGAAGCCACCCCCATGACCCCAAGGAAACACACCAAACAAGACAAAAACAGAGAGAAGAAGAGAAAGCTGATCAGGTTATGAGCTCCGTTGCATGGTGCTGGTGAACAGACCAATTTATGCC	chromosome 19; cyclin E1 (CCNE1) gene
M00853:35:000000000-AG8EE:1:1102:8135:19211_Cs_CON_consensus	TCTTCATCTGGATCCTGCAAAAACTAACATGAAGCCACCCCCATGACCCCAATGAAACACACCAAACAAGACAAAAACAGAGAGAAGAAGAGAAAGCTGATCAGGTTATGAGCTCCGTTGCATGGTGCTGGTGAACAGACCAATTTATGCC	chromosome 19; cyclin E1 (CCNE1) gene
M00853:35:000000000-AG8EE:1:1113:21390:24487_Cs_CON_consensus	TCTTCATCTGGATCCTGCAAAAACTAACATGAAGCCACCCCCATGACCCCAAGGAAACACACCAAACAAGACAAAAACAGAGAGAAGAAGAGAAAGCTGATCAGGTTATGAGCTCCGTTGCATGGTGCTGGTGAACAGACCAATTTATGCC	chromosome 19; cyclin E1 (CCNE1) gene
M00853:35:000000000-AG8EE:1:1104:25785:22003_Cs_CON_consensus	TCTTCATCTGGATCCTGCAAAAACTAACATGAAGCCACCCCCATGACCCCAAGGAAACACACCAAACAAGACAAAAACAGAGAGAAGAAGAGAAAGCTGATCAGGTTATGAGCTCCGTTGCATGGTGCTGGTGAACAGACCAATTTATGCC	chromosome 19; cyclin E1 (CCNE1) gene
M00853:35:000000000-AG8EE:1:2114:27298:22333_Cs_CON_consensus	TCTTCATCTGGATCCTGCAAAAACTAACATGAAGCCACCCCCATGACCCCAAGGAAACACACCAAACAAGACAAAAACAGAGAGAAGAAGAGAAAGCTGATCAGGTTATGAGCTCCGTTGCATGGTGCTGGTGAACAGACCAATTTATGCC	chromosome 19; cyclin E1 (CCNE1) gene
M00853:35:000000000-AG8EE:1:2105:24345:17446_HBV_3215	CGACCTTGAGGCATACTTCAAAGACTGTTTGCAGAAATATAACTGCAAGCATTTCAGAGGGCATTCCAGCAGTAGGCACTGTAAAGTTGCAAAGAGTTTTTGCCCCAGCAATTCCACTTCCAGCACTCAGTGTTTCAGAAGTACTTTAAC	RP11-1M19 on chromosome 9q34.11-34.3
M00853:35:000000000-AG8EE:1:1107:19522:10722_HBV_3215	CGACCTTGAGGCATACTTCAAAGACTGTTTATTTCTATTATTCTCTCCAGCACAAATGGACAGCAGAGTAACCTGGGGACACGGCACGCTGGGGCAGGGGACGTAGCAGAAACGGCACACTATCTTACACGCTTAGCAGGGCCCGCTCCGC	RP11-206I15 on chromosome 13
M00853:35:000000000-AG8EE:1:2103:5077:8484_HBV_3215	CGACCTTGAGGCATACTTCAAAGACTGTTTTCAAACTGCTGAATCAAAAGAAATGTTGAACTCCATGAGTTGAATGCACACGTACCAACGTCTGAACTCCAGTCACGTCCGCACAACTCGTATTCCGTCTTCTGCTTGAAAAAAAAAAATT	Pongo abelii BAC clone CH276-355F4 from chromosome unknown
M00853:35:000000000-AG8EE:1:2114:25079:12164_HBV_3215	CGACCTTGAGGCATACTTCAAAGACTGTTTGCCTGGGTATCACCAGCGGAAGCTGCAGAACAGCCACGATTGCTGCCTCTTCCTTCCTCTGGAAGCTTTGTCCCAGAGGCGGACCCACCAGCTGCCAGCCAGAGCTCTCCTGTATGAGATG	dedicator of cytokinesis 3 (DOCK3), chromosome x
M00853:35:000000000-AG8EE:1:2104:4179:18470_HK2100	GAAAAAGATTCATAGAAACAAACATGAACACCCACCATGTCTTGCCAAAGGTATTATATAAGAGGACTCTGGGAAACTCAGCTATTTCAACGACCTTCTATGAGGCGTACTTCAAGGACTGTGAGTTTAAAGACTGGGAGGCGTTGGGGGGAAAAAGTTGCATG	chromosome 16 clone RP11-105C20
M00853:35:000000000-AG8EE:1:1111:18341:21952_Cs_CON_consensus	CGACCTTGAGGCATACTTCAAAGACTGTGTAAGACCGTGTAAGACCGTGTGTAAGACTGTGTGTAAGACTGCTTGTGTAAGACTGCGTTTGTAAGACCGTGTGTAAGACTGCGTGTGTAAGACCATGCGTAAGGCCGTGTGTAAGACTGTG	RP11-193J6 on chromosome 1

read1
