# Supplementary material for: ChimericSeq: An open-source, user-friendly interface for analyzing NGS data to identify and characterize viral-host chimeric sequences
Source: PLoS One. 2017 Aug 22;12(8):e0182843. doi: 10.1371/journal.pone.0182843 (PMC5567911; doi:10.1371/journal.pone.0182843)
Supplement: S3 Fig — (PDF) [file pone.0182843.s005.pdf]

			SEQUENCE	Detected integration site	
145	1689	122M29S	ACCGACCTTGAGGCATACTTCAAAGACTGTGTATTTAAAGACTGGGAGGAGTTGGGGGAGGAGATTAAGCTAAAGGTCTTTGTACTAGATAGCTGTAGACATAAATTGGTCTGTTCACCAGCGACATGCAACGGAGCTCATAACCTGATCA	1 (Chr19)	
145	1689	132M19S	ACCGACCTTGAGGCATACTTCAAAGACTGTGTATTTAAAGACTGGGAGGAGTTGGGGGAGGAGATTAGGCTAAAGGTCTTTGTACTAGGAGGCTGTAGGCATAAATTGGTATGTTCACCAGCACCATGCAACGGAGCTCATAACCTGATCA	1 (Chr19)	
145	1689	132M19S	ACCGACCTTGAGGCATACTTCAAAGACTGTGTATTTAAAGACTGGGAGGAGTTGGGGGAGGAGATTAGGCTAAAGGTCTTTGTACTAGGAGGCTGTAGGCATAAATTGGTCTGTTCACCAGCACCATGCAACGGAGCTAATAACCTGATCA	1 (Chr19)	
145	1689	132M19S	ACCGACCTTGAGGCATACTTCAAAGACTGTGTATTTAAAGACTGGGAGGAGTTGGGGGAGGAGATTAGGCTAAAGGTCTTTGTACTAGGAGGCTGTAGGCATAAATTGGTCTGTTCACCAGCACCATGCAACGGAGCTCATAACCTGATCA	1 (Chr19)	
145	1689	132M19S	ACCGACCTTGAGGCATACTTCAAAGACTGTGTATTTAAAGACTGGGAGGAGTTGGGGGAGGAGATTAGGCTAAAGGTCTTTGTACTATGAGGCTGTAGGCATAAATTGGTCTGTTCACCAGCACCATGCAACGGAGCTCATAACCTGATCA	1 (Chr19)	read-8
145	1689	132M19S	ACCGACCTTGAGGCATACTTCAAAGACTGTGTATTTAAAGACTGGGAGGAGTTGGGGGAGGAGATTAGGCTGAAGGTCTTTGTACTAGGAGGCTGTAGGCATAAATTGGTCTGTTCACCAGCACCATGCAACGGAGCTCATAACCTGATCA	1 (Chr19)	
145	1689	132M19S	ACCGACCTTGAGGCATACTTCAAAGACTGTGTATTTAAAGACTGGGAGTAGTTGGGGGAGGAGATTAGGCTAAAGGTCTTTGTACTAGGAGGCTGTAGGCATAAATTGGTCTGTTCACCAGCACCATGCAACGGAGCTCATAACCTGATCA	1 (Chr19)	
145	1689	132M19S	ACCGACCTTGAGGCATACTTCAAAGACTGTGTATTTAACGACTGGGAGGAGTTGGGGGAGGAGATTAGGCTAAAGGTCTTTGTACTAGGAGGCTGTAGGCATAAATTGGTCTGTTCACCAGCACCATGCAACGGAGCTCATAACCTGATCA	1 (Chr19)	
145	1689	132M19S	ACCGACCTTGAGGCATACTTCAAATACTGTGTATTTAAAGACTGGGAGGAGTTGGGGGAGGAGATTAGGCTAAAGGTCTTTGTACTAGGAGGCTGTAGGCATAAATTGGTCTGTTCACCAGCACCATGCAACGGAGCTCATAACCTGATCA	1 (Chr19)	
185	1662	128M23S	ACTCTTGGACTCTCAGCAAAGTCAACGACCGACCTTGAGGCATACTTCAAAGACTGTGTGTTTAAAGACTGGGAGGAGTTGGGGGAGGAGATTAGGTTAAAGGTCTTTGTGCTAGGAGGCTGTAGGCACAAAGTGGTCTCTTCACCTGCCC		
145	1727	94M57S	AGACTGGGAGGAGTTGGGGGAGGAGATTAGGCTAAAGGTCTTTGTACTAGGAGGCTGTAGGCATAAATTGGTCTGTTCACCAGCACCATGCAACGGAGCTCATAACCTGATCAGCTTTCTCTTCTTCTCTCTGTTTTTGTCTTGTTTGGTG	1 (Chr19)	read-6
145	1676	138M13S	AGCAATGTCAACGACCGACCTTGAGGCATACTTCAAAGACTGTGTGTTTAAAGACTGGGAGGAGTTGGGGGAGGAGATTAGGTTAAAGGTCTTTGTACTAGGAGGCTGTAGGCATAAATTGGTCTGTTCACCAGCACCGGAGCAGCCCCAT		
97	1742	79M43S	AGGGGAGGAGATAAGGTTAAAGGTCTTTGTACTAAGAGGCTGTAGGCATAAATTGGTCTGTTCACCAGCACCATGCAACGGAGCTCATAACCTGATCAGCTTTCTCTTCTTCTCTCTGTTTT	1 (Chr19)	
97	1742	79M19S	AGGGGAGGAGATAAGGTTAAAGGTCTTTGTACTAGGAGGCTGTAGGCATAAATTGGTCTGTTCACCAGCACCATGCAACGGAGCTCATAACCTGATCA	1 (Chr19)	read-4
97	1742	79M43S	AGGGGAGGAGATAAGGTTAAAGGTCTTTGTACTAGGAGGCTGTAGGCATAAATTGGTCTGTTCACCAGCACCATGCAACGGAGCTCATAACCTGATCAGCTTTCTCTTCTTCTCTCTGTTTT	1 (Chr19)	
97	1745	3S76M43S	AGTGGAGGAGATAAGGTTAAAGGTCTTTGTACTAGGAGGCTGTAGGCATAAATTGGTCTGTTCACCAGCACCATGCAACGGAGCTCATAACCTGATCAGCTTTCTCTTCTTCTCTCTGTTTT	1 (Chr19)	
145	1703	143M8S	ATACTTCAAAGACTGTGTGTTTAAAGACTGGGAGGAGTTGGGGGAGGAGATTAGGTTAATGATCTTTGTACTAGGAGGCTGTAGGCATAAATTGGTCTGCGCACCAGCACCATGCAACTTTTTCACCTCTGCCTAATCATCTCATGCGACG		
145	1675	143M6S	CAGCGATGTCAACGACCGACCTTGAGGCGTACTTCAAAGACTGTGTGTTTAAAGACTGGGAGGAGTTGGGGGAGGAGATTAGGTTAATGATCTTTGTACTAGGAGGCTGTAGGCATAAATTGGTCTGCGCACCAGCACCATGCGACGTG		
145	1675	143M6S	CAGCGATGTCAACGACCGACCTTGAGGCGTACTTCAAAGACTGTGTGTTTAAAGACTGGGAGGAGTTGGGGGAGGAGATTAGGTTAATGATCTTTGTACTAGGAGGCTGTAGGCATAAATTGGTGTGCGCACCAGCACCATGCGACGTA		
97	1694	127M24S	CCTTGAGGCATACTTCAAAGACTGTGTATTTAAAGACTGGGAGGAGTTGGGGGAGGAGATTAGGCTAAAGGTCTTTGTACTAGGAGGCTGTAGGCATAAATTGGTCTGTTCACCAGCACCATGCAACGGAGCTCATAACCTGATCAGCTTT	1 (Chr19)	read-7
97	1694	127M24S	CCTTGAGGCATACTTCAAAGACTGTGTATTTAAAGACTGGGAGGAGTTGTGGGAGGAGATTAGGCTAAAGGTCTTTGTACTAGGAGACTGTAGGCATAAATTGGTCTGTTCACCAGCACCATGCAACGGAGCTCATAACCTGATCAGCTTT	1 (Chr19)	
73	1691	30M121S	CGACCTTGAGGCATACTTCAAAGACTGTCTTCAGTACTACATGAGGTAATACATAGAATACACTTGAAAAATGTTAGTGAGTCTCATGTAGTCACGTCCGCACATCTCGTATGCCGTCTTCTGCTTGAAAAAAAAAAAAAAACACTGACGT		
73	1691	30M120S	CGACCTTGAGGCATACTTCAAAGACTGTCTTCAGTACTACATGAGGTAATACATAGAATACACTTGAAAAATGTTAGTGAGTCTCATGTAGTCACGTCCGCACATCTCGTATGCCGTCTTCTGCTTGAAAAAAAAAAAAAAGAAAAACAAA		
73	1691	30M121S	CGACCTTGAGGCATACTTCAAAGACTGTGCACACCTGGGCACGGTCCTGGGTGCTGCTCACAGACACACTGCCTTTGGGTAATTCTCTTGGAAGGATTGCTTCCATGTACCTGGAGATGTCACTCAAGGAGGATGGAGAAAAGCAAATATC		
73	1691	30M121S	CGACCTTGAGGCATACTTCAAAGACTGTGGCAAAATAAATGGATGTTGTGTTAAAAAGAAAAAACCTAAACACTCAGAAAAAAAGAAAAATGTTGGCAGTACAAAGAGATATTGCCAGAAACACAAATATAGTGAGTCTTTAACATACTAT		
73	1691	32M119S	CGACCTTGAGGCATACTTCAAAGACTGTGGGTAACGAAAAGGGTTGGGATAAGTCAGGGAGAGCTAGGGTGGGGACAGTCTCTAAAGCTGTCTTCAATGAACGGAAAGAGGAGTGGGGAAAGGATTTAGAATCTATGGGGTCAGCTAGGTT		
73	1691	31M120S	CGACCTTGAGGCATACTTCAAAGACTGTGTAAGACCGTGTAAGACCGTGTGTAAGACTGTGTGTAAGACTGCTTGTGTAAGACTGCGTTTGTAAGACCGTGTGTAAGACTGCGTGTGTAAGACCATGCGTAAGGCCGTGTGTAAGACTGTG		
73	1691	31M119S	CGACCTTGAGGCATACTTCAAAGACTGTTTAATCAACTACTCCCACTGCTAATAATTTTTCACCTCACTGGTACTTATGATCATTTGACCTGTTTATTTCCCTCTATCCTCCAGTCACGTCCGCACATCTCGTATGCCGTCTTCTGCTTG		
73	1691	31M119S	CGACCTTGAGGCATACTTCAAAGACTGTTTAATCAACTTCTCCCACTGATAATAATTTTTCACCTCACTGGTACTTATGATCATTTGACCTGTTTATTTCCCTCTATCCTCCAGTCACGTCCGCACATCTCGTATGCCGTCTTCTGCTTG		
73	1691	31M119S	CGACCTTGAGGCATACTTCAAAGACTGTTTAATCAACTTCTCCCACTGCTAATAATTTTTAACCTCACTGGTACTTATGATCATTTGACCTGTTTATTTCCCTCTATACTCCAGTCACGTCCGCACATCTCGTATGCCGTCTTCTGCTTG		
73	1691	31M119S	CGACCTTGAGGCATACTTCAAAGACTGTTTAATCAACTTCTCCCACTGCTAATAATTTTTCACCTCACTGGTACTTATGATCATTTGACCTGTTTATTTCCCTCTATCCTCCAGACACGTCCGCACATCTCGTATGCCGTCTTCTGCTTG		
73	1691	31M119S	CGACCTTGAGGCATACTTCAAAGACTGTTTAATCAACTTCTCCCACTGCTAATAATTTTTCACCTCACTGGTACTTATGATCATTTGACCTGTTTATTTCCCTCTATCCTCCAGTCACGTCCGCACATCTCGTATGCCGTCTTCTGCTTG		
73	1691	31M120S	CGACCTTGAGGCATACTTCAAAGACTGTTTAATCAACTTTTCCCACTGCTAATAATTTTTCACCTCACTGGTACTTATGATCATTTGACCTGTTTATTTTCCTCTATCCTCCAGTCACGTCCGCACATCTCGTATGCCGTCTTCTGCTTGG		
73	1691	31M120S	CGACCTTGAGGCATACTTCAAAGACTGTTTAGTGGTCTCTTCACACGGCTGCACGTGACATGTGGTGCTGAAGACCCGGGACAGGAGGACTCCTTCAGGAGACCGGTCCCCTGTCCTTGCCCTCACTCCGTGAGGAGATCCACCTACAACC		
73	1691	32M119S	CGACCTTGAGGCATACTTCAAAGACTGTTTATAACCTAAGAAATAATTACACATATGTTATATATAAAAATGGGTCCTTAGAGATTGGCACGGCCGCACATATCGTATTCCGTCTTCTGCTTGAAAAAAAAAAAACACGTCTGAACTCCAGT		
73	1691	34M117S	CGACCTTGAGGCATACTTCAAAGACTGTTTATTTCTATTATTCTCTCCAGCACAAATGGACAGCAGAGTAACCTGGGGACACGGCACGCTGGGGCAGGGGACGTAGCAGAAACGGCACACTATCTTACACGCTTAGCAGGGCCCGCTCCGC		
73	1691	30M121S	CGACCTTGAGGCATACTTCAAAGACTGTTTCAGTTTAGGGAAGTCTGAGTATGAAGATCTTAATGTCTCTGTCTGGGAAGATGGCTCTCCTGGAGAGGCTGAGGTGGATATCAGTTCCTCCACTGCCTATAAAATAATCAAAACTGTTTCT		
73	1691	30M121S	CGACCTTGAGGCATACTTCAAAGACTGTTTCCATTTCCATTATACTCTTTTTTTTTTTTTTTTTTTTGAAAGGGGGCTTTCCCCTTCCCCCCAGGTAAAGGGGCGGGGGCCCATTTTGGGTCACTGTCAACCCCTCCCCCCTGGGTCCACCC		
73	1691	31M120S	CGACCTTGAGGCATACTTCAAAGACTGTTTGAAAACACACAGAGGAGAAAAAAGAATAAAAACCAACCAGCCAACCACACAACAGCAACAAAAGGCCAATTTAAAATAGGATTCACTTTATAAAAATCACCCTAACCCATCACGTCCGCAC		
73	1691	31M120S	CGACCTTGAGGCATACTTCAAAGACTGTTTGAAAAGAGAAAAGTCAGTAGAGAGAGGTAACAAACTTAAATATGTGCCAAGTCTAGAAACAAGAGACCAGGGGGATAAGGACCTTTCAAAATAAAATGCAAGATTTGAAAACTGATTGGCG		
73	1691	31M119S	CGACCTTGAGGCATACTTCAAAGACTGTTTGAAGAAACAGAACAAGCACTACAATTGGACTCAGATACAGCAGGGATGTTGAAATTATCAGACTGAGTTTTTAAAACAACTATGATTACTATACTAAAAACCTTAATTGATAATGCAAAT		
73	1691	32M118S	CGACCTTGAGGCATACTTCAAAGACTGTTTGCAGAAATATAACTGCAAGCATTTCAGAGGGCATTCCAGCAGTAGGCACTGTAAAGTTGCAAAGAGTTTTTGCCCCAGCAATTCCACTTCCAGCACTCAGTGTTTCAGAAGTACTTTAAC		
73	1691	32M119S	CGACCTTGAGGCATACTTCAAAGACTGTTTGCCTGGGTATCACCAGCGGAAGCTGCAGAACAGCCACGATTGCTGCCTCTTCCTTCCTCTGGAAGCTTTGTCCCAGAGGCGGACCCACCAGCTGCCAGCCAGAGCTCTCCTGTATGAGATG		
73	1691	33M117S	CGACCTTGAGGCATACTTCAAAGACTGTTTGCTATGACTTTAATTCCTTTACATTTTCTGAGGAGTGTTTTACTTCCAATTATGTGGTCAATTTTGAACTCTGAACTCCAGTCACGTCCGCACATCTCGTATGCCGTCTTCTGCTTGAAA		
73	1691	30M120S	CGACCTTGAGGCATACTTCAAAGACTGTTTTCAAACTGCTGAATCAAAAGAAATGTTGAACTCCATGAGTTGAATGCACACGTACCAACGTCTGAACTCCAGTCACGTCCGCACAACTCGTATTCCGTCTTCTGCTTGAAAAAAAAAAATT		
73	1691	30M120S	CGACCTTGAGGCATACTTCAAAGACTGTTTTGTCTGAAATAAGAATAGCAACCTCTGCTTTTTCTTTCTTTTTTATTTGCTCGATAGACCTTTGCTCGCCCAGTCACGTCCGCACATCTCGTATGCCGTCTTCTGCTTGAAAAAAAAAAA		
185	1750	65S61M25	CGCGTCAGGATTTGACTCCTCACTCAAAGTAGGTGTGTTTTTAGACTGTGTGGATTGTGGTGTGTAGATTAGGTTAAAGGTTAGTGTACTAGAATGCTGTAGGTATAAATTGGTCTGTTCACCAGCTCCGGAGCTGCTCCATAATGCCGAG		
145	1767	72S40M39	CGTGGGGCTTTCTTTCAAGGTTCTGGTATTCAAGACCTGGGGGATGTCGGGGGCGGAGTTAGGTTTACGGTTTTTGTGCTAGGAGGCTGTAGGCACAAATTGGTCTGTTCACGAGCCCCGGTGCAGCATCTTTCTGCCGAGGGAGCGCCGG		
137	1599	23S127M	CGTTGGCACTGCTCTTGACCTGGGCACGTCGCAGGGAAAGCACCGTGAACGGCCGCTAGGACTTGCCCAAGGGCTGACATAAGAGGACTCTTGGACTCTCAGCAATGTCAACGACCGACGTTGACGCATACTGCAAAGACTGTGTGTTTA		
145	1713	108M43S	GACTGTGTATTTAAAGACTGGGAGGAGTTGGGGGAGGAGATTAGGCTAAAGGTCTTTGTACTAGGAGGCTGTAGGCATAAATTGGTCTGTTCACCAGCACCATGCAACGGAGCTCATAACCTGATCAGCTTTCTCTTCTTCTCTCTGTTTT	1 (Chr19)	read-5
145	1713	108M43S	GACTGTGTATTTAAAGACTGGGAGGAGTTGTGGGAGGAGATTAGGCTAAAGGTCTTTGTACTAGGAGACTGTAGGCATAAATTGGTCTGTTCACCAGCACCATGCAACGGAGCTCATAACCTGATCAGCTTTCTCTTCTTCTCTCTGTTTT	1 (Chr19)	
145	1746	75M76S	GAGGAGATTAGGCTAAAGGTCTTTGTACTAGGAGGCTGTAGGCATAAATTGGTATGTTCACCAGCACCATGCAACGGAGCTCATAACCTGATCAGCTTTCTCTTATTCTCTCTGTTTTTGTCTTGTTTGGTGTGTTTCCTTGGGGTCATGG	1 (Chr19)	
145	1746	75M76S	GAGGAGATTAGGCTAAAGGTCTTTGTACTAGGAGGCTGTAGGCATAAATTGGTCTGTTAACCAGCACCATGCAACGGAGCTCATAACCTGATCAGCTTTCTCTTCTTCTCTCTGTTTTTGTCTTGTTTGGTGTGTTTCCTTGGGGTCATGG	1 (Chr19)	
145	1746	75M76S	GAGGAGATTAGGCTAAAGGTCTTTGTACTAGGAGGCTGTAGGCATAAATTGGTCTGTTCACCAGCACCATGCAAAGGAGCTCATAACCTGATCAGCTTTCTCTTCTTCTCTCTGTTTTTGTCTTGTTTGGTGTGTTTCCTTGGGGTCATGG	1 (Chr19)	
145	1746	75M76S	GAGGAGATTAGGCTAAAGGTCTTTGTACTAGGAGGCTGTAGGCATAAATTGGTCTGTTCACCAGCACCATGCAACGGAGCTCATAACCTGATCAGCTTTCTATTCTTCTCTCTGTTTTTGTCTTGTTTGGTGTGTTTCCTTGGGGTCATGG	1 (Chr19)	
145	1746	75M76S	GAGGAGATTAGGCTAAAGGTCTTTGTACTAGGAGGCTGTAGGCATAAATTGGTCTGTTCACCAGCACCATGCAACGGAGCTCATAACCTGATCAGCTTTCTCTTATTCTCTCTGTTTTTGTCTTGTTTGGTGTGTTTCCTTGGGGTCATGG	1 (Chr19)	
145	1746	75M76S	GAGGAGATTAGGCTAAAGGTCTTTGTACTAGGAGGCTGTAGGCATAAATTGGTCTGTTCACCAGCACCATGCAACGGAGCTCATAACCTGATCAGCTTTCTCTTCTTCTATCTGTTTTTGTCTTGTTTGGTGTGTTTCCTTGGGGTCATGG	1 (Chr19)	
145	1746	75M76S	GAGGAGATTAGGCTAAAGGTCTTTGTACTAGGAGGCTGTAGGCATAAATTGGTCTGTTCACCAGCACCATGCAACGGAGCTCATAACCTGATCAGCTTTCTCTTCTTCTCTCTGTTTTTGTCTTGTTTGGGGTGTTTCCTTTGGGTCATGG	1 (Chr19)	
145	1746	75M76S	GAGGAGATTAGGCTAAAGGTCTTTGTACTAGGAGGCTGTAGGCATAAATTGGTCTGTTCACCAGCACCATGCAACGGAGCTCATAACCTGATCAGCTTTCTCTTCTTCTCTCTGTTTTTGTCTTGTTTGGTGTGTTTCCTTGGGGTCATGG	1 (Chr19)	
145	1746	75M76S	GAGGAGATTAGGCTAAAGGTCTTTGTACTAGGAGGCTGTAGGCATAAATTGGTCTGTTCACCAGCACCATGCAACGGAGCTCATAACCTGATCAGCTTTCTCTTCTTCTCTCTGTTTTTGTCTTGTTTTGTGTGTTTCCTTGGGGTCATGG	1 (Chr19)	read-3
145	1746	75M76S	GAGGAGATTAGGCTAAAGGTCTTTGTACTAGGAGGCTGTAGGCATAAATTGGTCTGTTCACCAGCACCATGCAACGTAGCTCATATCCTGATCAGCTTTCTCTTCTTCTCTCTGTTTTTGTCTTGTTTGGTGTGTTTACTTGGGGTCATGG	1 (Chr19)	
145	1746	75M76S	GAGGAGATTAGGCTAAAGGTCTTTGTACTAGGAGGCTGTAGGCATAAATTGGTCTGTTCACCATCACCATGCAACGGAGCTCATAACCTGATCAGCTTTCTCTTCTTCTCTCTGTTTTTGTCTTGTTTGGTGTGTTTCCTTGGGGTCATGG	1 (Chr19)	
145	1746	75M76S	GAGGAGATTAGGCTAAAGGTCTTTGTACTAGGAGGCTGTAGGCATAAATTGGTCTGTTCATCAGCACCATGCAACGGAGCTCATAACCTGATCAGCTTTCTCTTCTTCTCTCTGTTTTTGTCTTGTTTGGTGTGTTTCCTTGGGGTCATGG	1 (Chr19)	
145	1746	75M76S	GAGGAGATTAGGCTAAAGGTCTTTGTACTAGGAGGCTGTAGGCATAAATTGGTCTGTTCTCCAGCACCATGCAACGGAGCTCATAACCTGATCAGCTTTCTCTTCTTCTCTCTGTTTTTGTCTTGTTTGGTGTGTTTCCTTGGGGTCATGG	1 (Chr19)	
145	1746	75M76S	GAGGAGATTATGCTAAAGGTCTTTGTACTAGGAGGCTGTAGGCATAAATTGGTCTGTTCACCAGCACCATGCAACGGAGCTCATAACCTGATCAGCTTTCTATTCTTCTCTCTGTTTTTGTCTTGTTTGGTGTGTTTCCTTGGGGTCATGG	1 (Chr19)	
145	1746	75M76S	GAGGGGATTAGGCTAAAGGTCTTTGTACTAGGAGGCTGTAGGCATAAATTGGTCTGTTCACCAGCACCATGCAACGGAGCTCATAACCTGATCAGCTTTCTCTTCTTCTCTCTGTTTTTGTCTTGTTTGGTGTGTTTCCTTGGGGTCATGG	1 (Chr19)	
81	1786	35M116S	GGCATAAATTGGTCTGTTCACCAGCACCATGCAACGGAGCTCATAACCTGATAAGCTTTCTCTTCTTCTCTCTGTTTTTGTCTTGTTTGGTGTGTTTCCTTGGAGTCATGGGGGTGGCTTCATGTTAGTTTTTGCAGGATCCAGATGAAGA	1 (Chr19)	
81	1786	35M116S	GGCATAAATTGGTCTGTTCACCAGCACCATGCAACGGAGCTCATAACCTGATCAGCTTTCTCTTCTTCTCTCTGTTTTTGTCTTGTTTGGTGTGTTTCATTGGGGTCATGGGGGTGGCTTCATGTTAGTTTTTGCAGGATCCAGATGAAGA	1 (Chr19)	read-1
81	1786	35M116S	GGCATAAATTGGTCTGTTCACCAGCACCATGCAACGGAGCTCATAACCTGATCAGCTTTCTCTTCTTCTCTCTGTTTTTGTCTTGTTTGGTGTGTTTCCTTGGAGTCATGGGGGTGGCTTCATGTTAGTTTTTGCAGGATCCAGATGAAGA	1 (Chr19)	
81	1786	35M116S	GGCATAAATTGGTCTGTTCACCAGCACCATGCAACGGAGCTCATAACCTGATCAGCTTTCTCTTCTTCTCTCTGTTTTTGTCTTGTTTGGTGTGTTTCCTTGGGGTCATGGGGGTGGCTTCATGTTAGTTTTTGCAGGATCCAGATGAAGA	1 (Chr19)	
145	1779	42M109S	GGCTGTAGGCATAAATTGGTCTGTTCACCAGCACCATGCAACGGAGCTCATAACCTGATCAGCTTTCTCTTCTTCTCTCTGTTTTTGTCTTGTTTGGTGTGTTTCCTTGGGGTCATGGGGGTGGCTTCATGTTAGTTTTTGCAGGATCCAG	1 (Chr19)	read-2
9	]-(1742	79M72S	GGGGGAGGAGATAAGGTTAAAGGTCATACCGGGAGAATTCAGCAGAGGCAAGATTGGAAA		
97	1742	79M19S	GGGGGAGGAGATAAGGTTAAAGGTCTTTGTACTAGGAGGCTGTAGGCATAAATTGGTCTGTTCACCAGCACCATGCAACGGAGCTCATAACCTGATCA	1 (Chr19)	read-4
97	1742	79M72S	GGGTGAGGAGATTAGGCTAAAGGTCTTTGTACTAGGAGGCTGTAGGCATAAATTGGTCTGTTCACCAGCACCATGCAACGGAGCTCATAACCTGATCAGCTTTCTCTTCTTCTCTCTGTTTTTGTCTTGTTTGGTGTGTTTCCTTGGGGTC	1 (Chr19)	
145	1682	132M18S	GTCAACGACCGACCTTGAGGCATACTTCAAAGACTGTGTGTTTAAAGACTGGGAGGAGTTGGGGGAGGAGATTAGGTTAAAGGTCTTTGTACTAGGAGGCTGTAGGCATAAATTGGTCTGTTCACCAGCACCGGAGCAGCCCCATCATGC		
145	1656	95S55M	GTGTTTTTTGTTTTTTTTTTTAATGATACGGCGACCACCGAGATCTACACTATAGCCTACACTCTTTCCTACACGACGCTCTTCCGATCTTGCACAACAGGACTCTTGGACTCTCAGCAATGTCAACGACCGACCTTGAGGCATACTTCA		
97	1659	7S124M	TGACAACAGGACTCTTGGACTCTCAGCAATGTCAACGACCGACCTTGAGGCATACTTCAAAGACTGTGTGTTTAAAGACTGGGAGGAGTTGGGGGAGGAGATTAGGTTAAAGGTCTTTGTACTAGGAGGCT		
97	1656	5S66M	TGCACAACAGGACTCTTGGACTCTCAGCAATGACAACGACCGACCTTGAGACATACTTCAAAGACTGTGTG		
97	1656	5S44M	TGCACAACAGGACTCTTGGACTCTCAGCAATGTCAACGACCGACCTTGA		
97	1659	8S143M	TGCACAACAGGACTCTTGGACTCTCAGCAATGTCAACGACCGACCTTGAGGCATACTTCAAAGACTGTGTATTTAAAGACTGGGAGGAGTATTGTGAGGCGATGAGGCTAAAGTACTTTGTACTAGTAGGCTGTAGGCATAAATTGGTCTG		
97	1659	8S143M	TGCACAACAGGACTCTTGGACTCTCAGCAATGTCAACGACCGACCTTGAGGCATACTTCAAAGACTGTGTATTTAAAGACTGGGAGGAGTTGGGGGAGGAGATTAGGCTAAAGGTCTTTGTACTAGGAGGCTGTAGGCATAAATTGGTCTG		
97	1659	8S143M	TGCACAACAGGACTCTTGGACTCTCAGCAATGTCAACGACCGACCTTGAGGCATACTTCAAAGACTGTGTATTTAAAGACTGGGAGGAGTTGGGGGAGGAGATTAGGCTGAAGGTCTTTGTACTAGGAGGCTGTAGGCATAAATTGGTCTG		
97	1659	8S143M	TGCACAACAGGACTCTTGGACTCTCAGCAATGTCAACGACCGACCTTGAGGCATACTTCAAAGACTGTGTATTTAAAGACTGGGAGTAGTTGGGGGAGGAGATTAGGCTAAAGGTCTTTGTACTAGGAGGCTGTAGGCATAAATTGGTCTG		
97	1659	8S143M	TGCACAACAGGACTCTTGGACTCTCAGCAATGTCAACGACCGACCTTGAGGCATACTTCAAAGACTGTGTATTTAACGACTGGGAGGAGTTGGGGGAGGAGATTAGGCTAAAGGTCTTTGTACTAGGAGGCTGTAGGCATAAATTGGTCTG		
97	1659	8S65M	TGCACAACAGGACTCTTGGACTCTCAGCAATGTCAACGACCGACCTTGAGGCATACTTCAAAGACTGTGTGTT		
97	1659	8S73M	TGCACAACAGGACTCTTGGACTCTCAGCAATGTCAACGACCGACCTTGAGGCATACTTCAAAGACTGTGTGTTTAAAGACT		
145	1659	8S84M	TGCACAACAGGACTCTTGGACTCTCAGCAATGTCAACGACCGACCTTGAGGCATACTTCAAAGACTGTGTGTTTAAAGACTGGGAGGAGTTG		
97	1659	8S94M	TGCACAACAGGACTCTTGGACTCTCAGCAATGTCAACGACCGACCTTGAGGCATACTTCAAAGACTGTGTGTTTAAAGACTGGGAGGAGTTGGGGGAGGAGA		
97	1659	8S96M	TGCACAACAGGACTCTTGGACTCTCAGCAATGTCAACGACCGACCTTGAGGCATACTTCAAAGACTGTGTGTTTAAAGACTGGGAGGAGTTGGGGGAGGAGATT		
97	1659	8S104M	TGCACAACAGGACTCTTGGACTCTCAGCAATGTCAACGACCGACCTTGAGGCATACTTCAAAGACTGTGTGTTTAAAGACTGGGAGGAGTTGGGGGAGGAGATTAGGTTAAA		
97	1659	8S106M	TGCACAACAGGACTCTTGGACTCTCAGCAATGTCAACGACCGACCTTGAGGCATACTTCAAAGACTGTGTGTTTAAAGACTGGGAGGAGTTGGGGGAGGAGATTAGGTTAAAGG		
97	1659	8S109M	TGCACAACAGGACTCTTGGACTCTCAGCAATGTCAACGACCGACCTTGAGGCATACTTCAAAGACTGTGTGTTTAAAGACTGGGAGGAGTTGGGGGAGGAGATTAGGTTAAAGGTCT		
97	1659	8S112M	TGCACAACAGGACTCTTGGACTCTCAGCAATGTCAACGACCGACCTTGAGGCATACTTCAAAGACTGTGTGTTTAAAGACTGGGAGGAGTTGGGGGAGGAGATTAGGTTAAAGGTCTTTG		
97	1659	8S118M	TGCACAACAGGACTCTTGGACTCTCAGCAATGTCAACGACCGACCTTGAGGCATACTTCAAAGACTGTGTGTTTAAAGACTGGGAGGAGTTGGGGGAGGAGATTAGGTTAAAGGTCTTTGTACTAG		
97	1659	8S121M	TGCACAACAGGACTCTTGGACTCTCAGCAATGTCAACGACCGACCTTGAGGCATACTTCAAAGACTGTGTGTTTAAAGACTGGGAGGAGTTGGGGGAGGAGATTAGGTTAAAGGTCTTTGTACTAGGAG		
97	1659	8S123M	TGCACAACAGGACTCTTGGACTCTCAGCAATGTCAACGACCGACCTTGAGGCATACTTCAAAGACTGTGTGTTTAAAGACTGGGAGGAGTTGGGGGAGGAGATTAGGTTAAAGGTCTTTGTACTAGGAGGC		
97	1659	8S124M	TGCACAACAGGACTCTTGGACTCTCAGCAATGTCAACGACCGACCTTGAGGCATACTTCAAAGACTGTGTGTTTAAAGACTGGGAGGAGTTGGGGGAGGAGATTAGGTTAAAGGTCTTTGTACTAGGAGGCT		
97	1659	8S124M19	TGCACAACAGGACTCTTGGACTCTCAGCAATGTCAACGACCGACCTTGAGGCATACTTCAAAGACTGTGTGTTTAAAGACTGGGAGGAGTTGGGGGAGGAGATTAGGTTAAAGGTCTTTGTACTAGGAGGCTAGATCGGAAGAGACGTCTG		
97	1659	8S136M	TGCACAACAGGACTCTTGGACTCTCAGCAATGTCAACGACCGACCTTGAGGCATACTTCAAAGACTGTGTGTTTAAAGACTGGGAGGAGTTGGGGGAGGAGATTAGGTTAAAGGTCTTTGTACTAGGAGGCTGTAGGCATAAAT		
97	1659	8S143M	TGCACAACAGGACTCTTGGACTCTCAGCAATGTCAACGACCGACCTTGAGGCATACTTCAAAGACTGTGTGTTTAAAGACTGGGAGGAGTTGGGGGAGGAGATTAGGTTAAAGGTCTTTGTACTAGGAGGCTGTAGGCATAAATTGGTCTG		
97	1656	5S56M90S	TGCACAACAGGACTCTTGGACTCTCAGCAATGTCAACGACCGACCTTGAGGCATACTTCAAGATCGGAAGAGCACAGTCTGAACTCCAGTCACACAGTGATCTCGTATGCCGTCTTCTGCTTGAAAAAAAAAAAAAACAAAAACAAATAGT		
145	1659	8S124M	TGCACAACAGGACTCTTGGACTCTCAGCAATGTCAACGACCGACCTTGAGGCATGCTTCAAAGACTGTGTGTTTAAAGACTGGGAGGAGTTGGGGGAGGAGATTAGGTTAAAGGTCTTTGTACTAGGAGGCT		
145	1659	8S73M	TGCACAACAGGACTCTTGGACTCTCAGCAATGTCAACGGCCGACCTTGAGGCATACTTCAAAGACTGTGTGTTTAAAGACT		
185	1659	8S131M1(	TGCACAACAGGACTCTTGGACTCTCAGCAATGTCAACGGCCGACCTTGAGGCATACTTCAAAGACTGTGTGTTTAAAGACTGGGAGGAGTTGGGGGAGGAGATTAGGTTAAAGGTCTTTGTACTAGGAGGCTGTAGGCAAGACCTGGTG		
145	1659	8S94M	TGCACAACAGGACTCTTGGACTCTCAGCAATGTCAGCGACCGACCTTGAGGCATACTTCAAAGACTGTGTATTTAAAGACTGGGAGGAGTTGGGGGAGGAGA		
145	1659	8S73M	TGCACAACAGGACTCTTGGACTCTCAGCAATGTCATCGACCGGACTTGAGGCATACTTCAAAGACTGTGTGTTTAAAGACA		
97	1659	8S143M	TGCACAACAGGACTCTTGGACTCTCAGCAATGTCCACGACCGACCTTGAGGCATACTTCAAAGACTGTGTATTTAAAGACTGGGAGGAGTTGGGGGAGGAGATTAGGCTAAAGGTCTTTGTACTAGGAGGCTGTAGGCATAAATTGGTCTG		
97	1659	8S84M	TGCACAACAGGACTCTTGGACTCTCAGCAATGTCCACGACCGACCTTGAGGCATACTTCAAAGACTGTGTGTTTAAAGACTGGGAGGAGTTG		
97	1659	8S143M	TGCACAACAGGACTCTTGGACTCTCAGCAATGTCGACGACCGACCTTGAGGCATACTTCAAAGACTGTGTATTTAAAGACTGGGAGGAGTTGGGGGAGGAGATTAGGCTAAAGGTCTTTGTACTAGGAGGCTGTAGGCATAAATTGGTCTG		
97	1659	8S73M	TGCACAACAGGACTCTTGGACTCTCAGCACTGTCAACGACCGACCTTGAGGCATACTTCAAAGACTGTGTGTTTAAAGACT		
97	1659	8S143M	TGCACAACAGGACTCTTGGACTCTCAGCGATGTCAACGACCGACCTTGAGGCGTACTTCAAAGACTGTGTGTTTAAAGACTGGGAGGAGTTGGGGGAGGAGATTAGGTTAATGATCTTTGTACTAGGAGGCTGTAGGCATAAATTGGTGTG		
73	1660	9S142M	TGCACAACAGGACTCTTGGACTCTCAGCGATGTCAACGACCGACCTTGAGGCGTATTTCAAAGACTGTGTGTTTAAAGACTGGGAGGAGTTGGGGGAGGAGAGTAGGTTAATGATCTTTGTACTAGGAGGCTGTAGGCATAAATTGGTCTG		
145	1659	8S63M	TGCACAACAGGACTCTTGGACTCTCAGTAATGTCAACGACCGATCTTGAGGCATACTTTAAAGACTGTGTG		
185	1659	8S57M	TGCACAACAGGACTCTTGGATTCTCAGCAATGTCAACGACCGACCTTGAGGCATACTTCAAAGAC		
185	1661	10S129M	TGCACAACATGACTCTTGGACTCTCAGCAATGTCAACGACCGACCTTGAGGCATACTTCAAAGACTGTGTGTTTAAAGACTGGGAGGAGTTGGGGGAGGAGATTAGGTTAAAGGTCTTTGTACTAGGAGGCTTTAGGCA		
